# The prolificacy of green transformational leadership in shaping employee green behavior during times of crises in small and medium enterprises: a moderated mediation model

**DOI:** 10.3389/fpsyg.2024.1258990

**Published:** 2024-02-23

**Authors:** Wafaa Mohammed Ahmed Zaid, Muhammad Zafar Yaqub

**Affiliations:** Department of Business Administration, Faculty of Economics and Administration, King Abdulaziz University, Jeddah, Saudi Arabia

**Keywords:** green transformational leadership, employee green behaviors, employee work engagement, employee self-esteem, SMEs, Saudi Arabia

## Abstract

Besides various other potent efforts to contain and consolidate, post-pandemic crisis management requires an adequate display of green transformational leadership. Green transformational leaders exhibit a formidable commitment to sustainability in making managerial choices and subsequently inspiring and motivating their employees to participate vigorously in the ensuing green initiatives. Such initiatives could profoundly help organizations adjust to shifting market situations, follow requirements, and preserve stakeholder trust. While making appeals to the central tenants of the transformation leadership theory, social exchange theory, and the social cognition theory, the study examines the impact of green transformational leadership on employees’ green behaviors during times of crises using employees’ self-esteem as a mediator and work engagement as the critical moderator. The relevant context of the study has been the small and medium enterprises of Saudi Arabia. Data from 232 SMEs employing less than 250 employees selected through convenience sampling was collected using structured questionnaires. After performing hierarchical regression modeling using SPSS 23, macro V4 model 58, and Amos 24, it has been found that green transformational leadership is positively associated with employees’ green behaviors, with self-esteem and work engagement acting as significant mediating and moderating conditions, respectively. Besides, providing firsthand empirical evidence on the impact of green transformational leadership on employees’ green behaviors, in unique industrial (SMEs), contextual (times of crises), and regional (Middle Eastern) settings, the study offers useful implications to the managers aspiring to enhance the sustainable performance of their firms through maturing green behaviors among their employees.

## 1 Introduction

Small and medium-sized enterprises (hereafter, SMEs) are businesses that typically exhibit a limited number of employees, assets, and revenues compared to large (corporate) firms. They make up about 95% of all businesses globally, contribute around 50% of the value created, and makeup 60–70% of all jobs in the majority of markets. They play a crucial role in driving economic growth, innovation, and job creation (Lekmat et al., 2018). By fostering entrepreneurship and innovation, SMEs help reduce unemployment rates and contribute to overall economic growth. In addition, SMEs are often more agile than larger corporations, allowing them to adapt more quickly to environmental changes. However, despite these merits, SMEs are more vulnerable to risk compared to large companies, especially during exigent times. Owing to their cardinal role in driving sustainable development, SMEs have attracted paramount interest from authorities, decision-makers, and researchers alike (Lekmat et al., 2018).

Joseph et al. (2022) contend that the current global landscape has been marked by a successive emergence of several crises including natural disasters, economic downturns, and pandemics. Firms, organizations, and institutions in the public as well as private domains need to find innovative ways to steer their entities effectively through the organizational crises stemming from environmental disruptions caused by black swan events such as COVID-19 (Li et al., 2020; McCartney et al., 2021; Clauss et al., 2022; Wu and Ho, 2022). These crises have had profound impacts on organizations, especially SMEs which often lack the requisite resources and infrastructure to cope with such challenges (Alenazi and Alanazi, 2023). A state of crisis can destabilize any organization and can significantly truncate employee productivity and efficiency, especially in SMEs where unusual disruptions caused, for example, by black swan events such as Covid-19, could necessitate a need for massive re-structuring or even shutdown of operations apart from the financial losses. Besides economic consequences, these crises also take a significant toll on the emotional and mental health of the SME owners, managers, employees, or other stakeholders making decision-making and/or the much-needed agility of response even more difficult (Klyver and Nielsen, 2021). The majority of SMEs reported an explicit reduction of work activity and production during COVID-19 times, which continued even post-pandemic. Hence, it is a cardinal obligation of organizational leaders to devise effective strategies to combat a plethora of challenges stemming from such environmental disruptions to keep up their commitment to sustainable actions being one of them (Sarkar and Clegg, 2021; Clauss et al., 2022).

Like all entities, SMEs are also vulnerable to crises such as natural disasters, economic downturns, and pandemics, which can profoundly impact their survival and success (Shafi et al., 2020). Though SMEs have been continuously evolving, their ability to combat risk is still inferior to that of large firms (Shafi et al., 2020). Besides other instruments, green behavior (hereafter, GBs) has been revealed as critical for organizational sustainability and could profoundly augment organizational performance, reputation, and stakeholder trust during tempestuous times (Singh et al., 2020). Many researchers have attested to the impact of GBs on firm performance during testing times. For example, Alraja et al. (2022) and Marditama and Yusoff (2023) contend that GBs can assist SMEs in recovering from crises, allowing them to re-function and regenerate revenue. Ionescu (2021) asserts that GBs ameliorate infrastructure, ecosystems, and community resilience. Ecosystem restoration and conservation increase biodiversity, which benefits humans and lessens risk. Alnemer et al. (2023) described that GBs during a crisis can create new green sector jobs and reduce dependence on non-renewable resources and hence, contribute significantly to economic stability. Green methods minimize operational expenses, making businesses more robust to economic shocks. Hence, any aspect of green behavior improvement at the organizational, team, or employee levels can improve an organization’s (sustainable) performance.

GBs, by fostering environmental consciousness and responsibility in employees, could contribute significantly to the sustainability performance of firms. GBs, by enabling innovation and growth, could also ensure long-term success (Roscoe et al., 2019). GBs become especially pivotal during exigent times (like Covid-19) to keep up green passion and commitment; hence, the green transformational leadership (hereafter, GTL) needs to find efficacious means of culminating, maturing, and sustaining such behaviors among employees during these testing times. Bakker et al. (2023) and Khan (2023) have elaborated upon the effect of green and transformational leadership on GBs, specifically how it motivates followers to reach their fullest potential in fostering transformation in an organization. GTLs are renowned for their charisma, vision, and capacity to empower and inspire their adherents to exceed performance expectations (Kim et al., 2021). Singh et al. (2020) also contend that GTL could profoundly motivate followers to achieve green goals, that could enable firms to add/enhance (green) value. This leadership style is crucial for ameliorating pro-environment workplace behaviors wherein employees are more likely to take green steps when GTLs interact/communicate cogently. As such, GTLs can inspire employees to adopt as well as stay committed to society and/or community-friendly practices. Even though a host of studies (e.g., Zhu et al., 2020; Çop et al., 2021; Ionescu, 2021; Agrawal and Pradhan, 2023; Ali et al., 2023; Alnemer et al., 2023; Zacher et al., 2023) have examined the efficacy of GBs in augmenting social and environmental performance, and the role of GTL in directly and or indirectly fostering GBs, in a variety of contexts, yet there exists a dearth of literature encompassing the mediating and contextual conditions that could enable or impede GTLs’ efforts to enhance EGBs for superior sustainability outcomes, especially during crises times. This research, while using transformational leadership theory, social exchange theory, and the social cognition theory as the underpinning theoretical frameworks, makes up for this deficiency in the research by examining the direct as well as indirect role of GTL in maturing GBs, while taking employee self-efficacy as mediating and employee work engagement (hereafter EWE) as the critical moderating contingency in the context of SMEs operating in an emerging economy (i.e., Saudi Arabia).

SMEs are increasingly considered essential to developing countries’ economic growth and prosperity (Tukamuhabwa et al., 2021). They are also contributing significantly to Saudi Arabia’s GDP by playing a crucial role in adding value and diversity to the economy. They typically hold less than 249 employees with a capacity of SAR 200 million in average revenue (Alenazi and Alanazi, 2023). As Saudi Arabia continues to grow exponentially on the socio-economic fronts, both in the domestic as well as global markets, SMEs are now one of the substantial contributors toward the materialization of the overarching Vision 2030 of Saudi Arabia which centers upon boosting socio-economic performance through ameliorating sustainability, employment opportunities, innovation, and technological developments in the country (Nurunnabi, 2020; Shafi et al., 2020). Alghamdi and AlKhayyat (2020) outline several benefits of SMEs and their crucial role over the years in the diversification of the Saudi economy with reference to creating job opportunities, innovation, social transformation, and regional development. Abid and Alotaibi (2020) also contend that SMEs are significant job creators, providing employment opportunities for the growing Saudi population. SMEs’ contributions to the diversification of the Saudi economy have progressively reduced the country’s reliance on oil revenues. This aligns well with Saudi Arabia’s Vision 2030, which aims to create a more sustainable and diversified economy. Consonantly, SMEs in Saudi Arabia, aided by the government’s support, consistently enhance their sustainability performance by fostering green orientation, passion, and action at the macro, meso, and micro levels (Naushad, 2021). This study focuses more on the micro (employee) level green behavior.

Comprehending the factors that drive EGBs in SMEs during crises is crucial for the long-term success and even survival of the firms as well as their leaders (Farahnak et al., 2020; Mohtady Ali et al., 2023), where the success of GTLs in successfully steering their organizations through troubled waters depends on their ability to inspire and motivate their followers to stay committed to green behaviors to preserve and/or maximize sustainability gains. Though researchers from various disciplines have contributed to maturing the scholarly discourse in this domain, significant research gaps still exist. To contribute to bridging some of these gaps in the literature, the current study seeks to answer the following research questions.

1. Does GTL, as an antecedent, drives EGBs in SMEs during crises?
2. Does FSE play a significant mediating role in the association between GTL and EGBs?
3. Does EWE play a significant moderating role in GTL-FSE linkage? FSE-GBs linkage?

The intended contributions of this research are manifold. First, it will enhance our understanding of the (psychological) mechanisms (i.e., FSE) through which GTL influences EGBs. Du and Yan (2022) explain that GTL is a relatively new aspect in the field of green management, in which there exists a profound emphasis on green motivation, green intellectual activation, and green care concerns that significantly contribute to the formation of the green vision for the employees inspiring them to work toward the attainment of green objectives. Second, by taking EWE as a critical moderating condition, the study would enhance our understanding of the contextual contingencies enabling or impeding GTLs’ quest to inculcate GBs among employees. Another contribution of this study could be traced back to its use of Middle Eastern data that not only could provide new insights into the dynamics of sustainability in this unique geographical context but would also help in cementing the generalizability of etic theories developed in the West to the disparate regional or cultural settings. As for the managerial contributions, the newfangled model would provide significant insights to the managers, leaders, and entrepreneurs on how to secure/enhance sustainability outcomes during exigent times through fostering EGBs enabled by GTL, FSE, and EWE. The study may offer insights into how SMEs could improve their resilience and sustainability during crises by enabling the roles that GTL, FSE, and ESE could play in nurturing EGBs.

The remainder of the paper is organized like this. The next section presents a detailed review of the literature, along with research gaps and the ensuing hypotheses of the study. Section 3 discusses research methods. The fourth section rolls out the results. The fifth section presents a discussion based on these results. The final section concludes the entire discussion while highlighting theoretical/ managerial contributions, limitations, and avenues for future research.

## 2 Literature review and hypotheses of study

Contemporary literature presents two opposing perspectives on SMEs’ attitudes toward environmental practices. First, compared to the larger enterprises, SMEs are less likely to adopt environmental practices and view social responsibility as a burden or even a threat as it may require significant investments in upgrading technologies, infrastructure, compliance, etc. (Koirala, 2018; Purwandani and Michaud, 2021; Majali et al., 2022). In contrast, certain factors could encourage SMEs to adopt environmentally friendly practices more easily. For example, small enterprises frequently combine ownership and management, so if the owner-manager is convinced of sustainable outcomes, getting everybody else on board becomes easy and swift. According to Clauss et al. (2022), SMEs could contrive business model innovations more swiftly to gain the much-needed strategic flexibility as a potent instrument to combat disruptions emanating from COVID-19, at least in the short run. Besides, cost savings, reputation effects, and consumer demands for green products are some other factors that induce green behaviors in SMEs, though such antecedents are more instrumental for larger firms (Liu et al., 2021; Majali et al., 2022). Mo et al. (2022b) and Ngo (2022) maintain that big companies have more elaborate structures that enable them to integrate green behaviors through robust leadership networks and the paradigms of culture formation. Big markets being served by these big companies also have particular incentives in green marketing and their influence on consumer green behaviors is relatively paramount (Wang et al., 2023). Consequently, big companies seem to have a strong incentive to integrate green initiatives. Nevertheless, there has been a continuous upsurge in the adoption of green behaviors by SMEs (Purwandani and Michaud, 2021; Thomas et al., 2022) and so has been in the research investigating the dynamics of EGBs in SMEs.

Besides other antecedents, the efficacy of GTL in maturing EGBs has been examined by different scholars through diverse theoretical lenses. Most of them have used efficacious underpinning frameworks like the theory of planned behavior, social cognition theory, social exchange theory, self-determination theory, ability-motivation-opportunity theory, etc., in contriving cause and effect relationships among a host of determinants of EGSs. Most scholarly theories associate GTL approaches with the motivation of workers and the adoption of engagement strategies that inspire employees to participate in innovative initiatives that underpin green transformation. Sobaih et al. (2022) and Ali et al. (2023) underscored that transformational leadership focuses mainly on inspiring employees and driving their behaviors toward environmental and green initiatives, thereby enhancing environmental performance. The culture of sustainability is also conceptualized as an outcome of GTL approaches to organizational management. Current frameworks of CSR initiatives are anchored on the attempts of organizational leaders to establish green resource management and sustainability programs within organizations (Sobaih et al., 2022). It would be challenging to achieve this transformation without deliberate efforts to change the behaviors of workers. Hence, the role of GTL is pivotal in this regard. We have used transformational leadership theory augmented with social exchange theory and the social cognition theory to conceptualize and later empirically substantiate our model that centers upon the direct as well as mediated effect of GTL on EGBs under varying permutations of EWE.

### 2.1 The underpinning theories

#### 2.1.1 Transformational leadership theory

The foundation of TL theory is definition of the TL as one who is able to separate followers from their trivial preoccupations and unite them around a common purpose to accomplish what they never thought was possible. TL envisages a leader who has the ability to detach followers from their preoccupations and rally them around a common goal. Bass (1985) created a typology of leadership characteristics that may be broadly classified into two categories: transactional leadership and transformational leadership. According to Bass’s (1985) interpretation of TL theory, which combined many aspects of prior leadership theories, transformational leadership requires inspiring and motivating followers to realize their greatest potential. Therefore, transformational leaders might be identified by their capacity for foresight, proficiency in communication, and ability to inspire followers to accomplish their goals (Bakker et al., 2023). They offer their followers encouragement and guidance, inspire them to think creatively and foster a culture that values working together. TL involves a variety of leader behaviors that have the potential to “transform” followers and positively influence their attitudes and actions. These behaviors consist of idealized influence, inspirational motivation, intellectual stimulation, and individualized care (Nohe and Hertel, 2017). Idealized influence refers to a leader’s capacity to serve as a role model for his/her adherents and earn their esteem and trust. Inspirational motivation is the capacity of a leader to inspire and motivate his or her adherents to accomplish higher levels of performance. Intellectual stimulation entails a leader’s capacity to encourage adherents to think creatively and query presuppositions. Individualized consideration is the capacity of a leader to provide individualized support and counseling to their adherents. TLs induce their followers to take ownership of their work and develop a sense of responsibility toward their co-workers and the organization, encouraging their followers to take the initiative, be proactive, and participate in team activities (Casu et al., 2021). Therefore, transformational leadership theory has been deemed an efficacious framework to account for the factors that drive SMEs to achieve the best practices and behaviors (including EGBs) to ensure their survival and sustainability during testing times.

#### 2.1.2 Social exchange theory

Social Exchange Theory (SET) is the foundation for numerous sociological and psychological theoretical frameworks. Homans (1961) contributed significantly to the development of this theory. Homans’ (1961) foundation is grounded in the premise of social behavior and how each party responds to the other based on the “sub-institutional” level of interaction. The outcome of the process is significantly influenced by the actions of both parties. Regarding the psychological aspect, Ahmad et al. (2023) contend that people engage in exchange relationships as a consequence of their interactions with others. People learn by progressively imitating the attitudes, values, and behavior of influential role models in their surroundings (Bandura, 1977). Chen et al. (2022) asserted that “green” behavior (GB) is frequently the result of “green practices” and that self-esteem plays a significant role in the process, as self-esteem can motivate employees to promote “green practices.” Additionally, self-respect plays a crucial role in the process by which “green” practices typically result from “green” conduct. Using this framework as a guide, it is possible to identify two distinct psychological processes as the means by which transformative leadership actions could ameliorate green behavior among employees. The first mechanism is predicated on the premise that GTL positively influences the internal conceptualization of the individual’s self-esteem, which in turn contributes to green employee behavior (Ahmad et al., 2021). This argument posits that the impact of transformational leadership on green behavior is merely the result of positive follower behaviors. However, transformational leadership’s long-term effects may involve more complex (bidirectional) relational exchange processes between leaders and followers. The second reason for the connection between EGB and GTL is that it emphasizes leaders’ and followers’ relational interactions. Transformational leaders and adherents engage in a high-quality relationship from this perspective. In order to recompense their GTLs’ exemplary behavior, followers engage in more greenly conscious actions (Farrukh et al., 2022). Consequently, we have employed SET as a relevant framework to examine the factors that drive EGBs in SMEs during times of crisis.

#### 2.1.3 Social cognition theory

Social cognitive theory (hereafter, SCT) has been extensively used in several studies that sought to establish how behavioral orientations are crucial in establishing green practices. He et al. (2021) used the SCT to explore the nexus between context, behavior, and performance in relation to green practices in organizations. He et al. (2021) underscored that the positive relationships between sustainability actions and employees’ performance have a strong dependence on social identification perspectives and behavior modeling orientations. From the lens of SCT, managers and leaders have the proclivities to establish green behaviors and the GTL becomes a critical enabler in the process. GTLs focus on mainstreaming social responsibility against the prevailing challenges of pollution and climate change (Yaqub et al., 2023). The supervisory role to encourage and support employees in pro-environmental activities is continually emphasized by GTLs who value sustainability (Ali et al., 2023). The optimization of resources is established as a goal and the trajectory of behavior performance and change is established within an organization. Al-Swidi et al. (2021), while emphasizing a nexus of employees’ activities and the behavioral inclinations in an organization, also underscore the essence of establishing cultures that resonate with intended actions and outcomes. Other researchers deviated from the traditional theories. For example, Mo et al. (2022a) and Ngo (2022) on “shaping employee green behavior” applied a multilevel approach with the Pygmalion effect from the theoretical and conceptual perspectives. Wang et al. (2023) acknowledge that the theoretical roots of studies on green behaviors and leadership are still lacking, and more in-depth analysis is required in the future. We have complemented TL theory and SET with SCT to explore the agency of social interactions in GTL’s inducement of EGBs during tempestuous times.

### 2.2 The conceptual model and the study constructs

**Figure 1:**
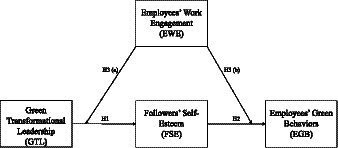
The conceptual model.

Figure 1 presents the conceptual model of our study. Making an appeal to the TL theory, SET, and SCT, it has been hypothesized that GTL affects EGBs through enhancing FSE. Further, the impact of GTL on FSE and the consequent impact of FSE on EGBs is moderated by the EWE.

The next section discusses the nature, scope, and relevance of the constructs that make our conceptual model.

#### 2.2.1 Employee green behavior during crisis – the outcome

EGBs include ensuring compliance with the organization’s standards, modifying work practices to incorporate the selection of responsible alternatives, and developing environmentally responsible products and procedures, is required (Zacher et al., 2023). Research on employee green behavior (GB) in the workplace has typically regarded it as a voluntary action. Nevertheless, organizational psychologists acknowledge that not all EGB is considered discretionary (Norton et al., 2015). Employees have the option of going beyond what is required of them by the company in terms of their environmental behavior. Katz et al. (2022) described the notion of voluntary GB that corresponds closely with the concepts of contextual performance and extension of corporate citizenship behavior. These concepts refer to actions that influence the organizational, social, and psychological environments in which task performance takes place. Voluntary GBs signify actions that foster an environment conducive to task performance. According to Chaudhary (2020), by establishing green positions and responsibilities, GB is indispensable for businesses seeking to improve their green performance.

During a crisis, SMEs often face financial challenges and must cut costs. EGBs, such as reducing energy consumption, minimizing waste, and using resources efficiently, can lead to significant cost savings for the company. These savings can help SMEs maintain the performance levels of their operations and even invest in other areas to overcome the crisis (Roscoe et al., 2019). Yong et al. (2020) explained that companies that demonstrate a sublime commitment to green and environmentally responsible practices can improve their reputation among customers, suppliers, and other stakeholders, particularly during a crisis, where a positive reputation can help SMEs maintain customer loyalty, attract new clients, and even secure funding or support from external sources. At the same time, Raza et al. (2021) proposed that encouraging EGBs can foster a sense of shared responsibility and purpose among staff members, leading to increased employee engagement and motivation, which is particularly important during a crisis when morale may be low, and employees need to work together to overcome challenges. Therefore, it is essential for SMEs to encourage and support EGBs, especially during times of crisis.

#### 2.2.2 Green transformational leadership – the antecedent

GTL, which amalgamates green and transformation leadership, is considered a vital approach in today’s rapidly changing world, as it emphasizes the need for organizations and leaders to adopt sustainable practices and promote environmental stewardship creating a positive impact on the environment and society. In a recent study, Liu and Yu (2023) found a positive impact of GTL in inducing OCBs among employees. Chen and Wu (2022) and Khan (2023) contend that green leadership could profoundly impact EGBs if efficacious green human resource practices are enabled. Ahmad et al. (2022) explain that GTLs prioritize environmental concerns and integrate them into their decision-making processes, promoting long-term sustainability by encouraging organizations to adopt eco-friendly practices that ensure that businesses remain viable and competitive in the long run as they adapt to the increasing demand for sustainable products and services. Younis and Hussain (2023) recently attested to the instrumentality of GTL in contriving a stronger green climate. Zhang et al. (2020, 2021) further added that GTLs inspire and motivate their employees to embrace sustainable practices and contribute to the organization’s environmental goals. They create a culture of environmental responsibility, where employees feel empowered to make a difference and take pride in their organization’s commitment to sustainability. This, in turn, leads to increased job satisfaction, higher retention rates, enhanced self-efficacy, and improved overall performance. Mittal and Dhar (2016) and Al-Ghazali et al. (2022), in a tourism industry context, found GTL to be positively associated with green creativity. Kusi et al. (2021) contend that organizations with strong sustainability practices perform better financially. GTL contributes to this by reducing operational costs through resource efficiency, attracting environmentally conscious customers, and mitigating risks associated with environmental regulations and potential reputational damage. The green makeover of organizations requires employees to not only work in ways that are better for the environment as part of their jobs but also to go beyond their own tasks and take the initiative to help the organization reach its goals (Darvishmotevali and Altinay, 2022; Du and Yan, 2022).

Tosun et al. (2022) posit that every organization must adopt the green approach to ensure that it is able to survive crises and boost organizational business performance even more. GTL steps up with deeper initiatives that support the green objectives and social issues that further support green behavior by choice (Li et al., 2020). Singh et al. (2020) enforced the view that it is mandatory for SMEs to adopt GTL to boost performance and creativity. Kwarteng et al. (2023) clearly established a link between transformational leadership, SE, and EGBs.

#### 2.2.3 Follower’s self-esteem – the mediator

According to identity theory, identities are an essential component of the self and are understood to be an individual’s internal understanding of their positions and designations in a variety of social settings (Cui et al., 2021). According to Markowski and Serpe (2021), the degree to which individuals evaluate themselves favorably or unfavorably signifies their self-esteem. According to Haider et al. (2019), followers with high self-esteem are likelier to have a positive self-image and engage in online community-building activities. According to Arokiasamy et al. (2022), leaders and employees can reduce their intentions to abandon their employment and implement tools, programs, and policies to support these crucial actions by boosting the self-esteem of their followers. Chen et al. (2022) claimed that “green” behavior is frequently the consequence of “green practices,” and that self-esteem plays a significant role in the process, where self-esteem can drive employees to promote “green” practices.

According to Pradies et al. (2021), self-esteem plays a crucial role in SMEs during times of crisis, as it can have a significant impact on both the organization’s overall performance and the conduct of its employees. Leaders and employees with a robust sense of self-worth can make more confident decisions in times of crisis. This confidence can aid organizations in effectively navigating challenging circumstances and generating innovative solutions to their problems. Raza et al. (2021) explained that individuals with a strong sense of self-worth are more likely to be dedicated to their work and take pride in their contributions, thereby increasing employee engagement. This may lead to increased productivity and enhanced business outcomes. Dahleez et al. (2022) emphasized that employees with high self-esteem are more likely to trust their colleagues and collaborate effectively during times of crisis. This can lead to enhanced problem-solving and decision-making, as well as a heightened sense of organizational cohesion. Another study by Zheng et al. (2022) presented findings relating to companies that cultivate a culture of high self-esteem, which is more likely to retain employees during challenging times. During a crisis, employees who feel valued and confident in their abilities are less likely to seek employment elsewhere. It is essential to maintain open communication, provide regular feedback and recognition, and provide opportunities for professional development and growth in order to support self-esteem within an organization during a crisis (Zheng et al., 2022). By nurturing a positive work environment that values and supports employees, businesses can navigate crises more effectively and emerge strongly from them.

#### 2.2.4 Employee work engagement – the moderator

Employee engagement is defined as the intellectual and emotional commitment to the organization demonstrated by the level of effort evidenced by workers in the performance of their duties. Saks (2006) extended Schaufeli et al. (2002) model by incorporating three dimensions of engagement: cognitive, affective, and behavioral (Kwon and Kim, 2020). Iqbal et al. (2021) explain that cognitive engagement indicates that an employee actively considers the information being communicated to him/her and makes connections to prior knowledge and experiences. Emotional engagement involves employees feeling motivated and enthusiastic about the subject matter, as well as a sense of belonging to the learning community. The behavioral dimension focuses on an individual’s actions and behaviors while engaged in their labor or tasks (Schaufeli et al., 2002).

Employee engagement theory is characterized by instilling feelings that allow the employees to challenge and support themselves through self- and external motivation that would lead to maximization of staff satisfaction and productivity (Saks and Gruman, 2014). According to this theory, organizations with high worker motivation and loyalty benefit from employee engagement through enhanced satisfaction and intellectual development (Kahn, 2010; Huang et al., 2022). Kahn (1990) identified meaningfulness, safety, and availability as the three primary characteristics that have the most significant impact on employee engagement. Highly engaged employees are more likely to be committed to the organization and its stakeholders and to engage in crisis-related work behaviors. Ababneh (2021) described the positive effect of employee engagement on the EGBs, in which high levels of engagement enhanced employees’ (green) performance within an organization. Zheng et al. (2022) conducted a study on SMEs in China during the COVID-19 pandemic and discovered that employee engagement positively moderated the relationship between leadership and EGBs.

### 2.3 Hypotheses of study

#### 2.3.1 GTL and FSE

According to Awan et al. (2022), GTL promotes green behaviors and practices in an organization wherein it emphasizes a strategy that centers on fundamental ideologies and principles of green transformation. FSE, which denotes employees’ overall sense of self-worth or personal value, can be affected by the quality of the followers’ social interactions and/or exchanges with their leadership (Kang, 2019). Awan et al. (2022), while examining the effects of GTL on the environmental performance of SMEs, found that. GTL significantly moderated the relationship between green capability and green innovation, highlighting GTL’s potential role in empowering employees despite the fact that environmental performance was the primary focus of this study. This can increase the self-esteem of adherents, reinforcing the premise that GTL has a positive effect on FSE. Srour et al. (2020) investigated the influence of GTL on the self-esteem of Egyptian IT personnel wherein the results suggested that GTL had a positive impact, thereby increasing FSE as they become more aligned with the green practices of their organizations. Begum et al. (2022) provided additional support by examining, via cognitive processes, the role of GTL in promoting green innovation, which had shown a significant impact on FSE and engagement in this study. Besides, Chen et al. (2022) discovered a significant positive relationship between TL and SE, indicating that transformational leadership, particularly when centered on green behavior, can subsequently boost FSE. Finally, Hameed et al. (2021) investigated the connection between GTL and SE and demonstrated a positive association between the two constructs. In consonance with the aforementioned arguments, we hypothesize.

*H1*: GTL has a significant positive association with FSE.

#### 2.3.2 FSE and EGBs during the crisis

According to Chen et al. (2022), employees with higher self-esteem are more likely to engage in green behaviors, such as recycling, conserving energy, and supporting environmentally friendly policies. This is because individuals with high self-esteem tend to have a stronger sense of personal responsibility and are likelier to believe that their actions can make a difference in addressing environmental issues. Moreover, Cheema et al. (2020) stated that employees with high self-esteem are more likely to be intrinsically motivated to engage in green behaviors, as they derive satisfaction from acting in accordance with their values and beliefs.

During times of crisis, such as the COVID-19 pandemic, employees’ self-esteem has been significantly affected due to increased job insecurity, financial stress, and changes in work routines. Srivastava and Gupta (2022) found that employees with higher self-esteem were more resilient in the face of crisis-related stressors and were more likely to maintain their green behaviors. This resilience can be attributed to the fact that individuals with high self-esteem have a stronger sense of self-efficacy and are more confident in their ability to cope with challenges and adapt to new situations. In contrast, employees with low self-esteem may be more susceptible to the negative effects of crisis-related stressors, which can lead to a decrease in their green behaviors. According to Gkargkavouzi et al. (2019), individuals with low self-esteem are more likely to experience feelings of helplessness and powerlessness in the face of environmental problems, which can result in a reduced sense of personal responsibility and a lower likelihood of engaging in green behaviors. Furthermore, employees with low self-esteem may prioritize their immediate needs and concerns over long-term environmental goals, particularly during times of crisis when resources are scarce and survival instincts are heightened.

Touma (2021) proposed that organizations can implement various strategies to promote employees’ green behaviors during crises to enhance self-esteem. One such strategy is to provide employees with opportunities for skill development and training, which can help them feel more competent and confident in their abilities. Additionally, organizations can foster a supportive work environment by encouraging open communication, recognizing employees’ achievements, and providing constructive feedback. This can help employees feel valued and respected, which in turn could boost their self-esteem and motivation to engage in green behaviors (Touma, 2021). Besides, organizations can leverage the power of social influence by promoting a strong green culture and encouraging employees to serve as role models for their peers. Chaudhary (2020) found that employees who perceived their colleagues as engaging in green behaviors were more likely to adopt similar behaviors themselves. This social influence effect was particularly strong among employees with high self-esteem, as they were likelier to internalize their peers’ environmental values and norms. In line with these arguments, we hypothesize.

*H2*: FSE has a significant and positive association with EGBs during the *crisis.*

#### 2.3.3 The mediation effect hypothesis

According to Liu et al. (2021), GTL emphasizes social responsibility in organizational practices and, hence, during times of crisis, could play a crucial role in fostering a sense of resilience and adaptability among employees, contributing to increased self-esteem and environmentally conscious behaviors. Srour et al. (2020) stated that GTL’s capacity to inspire and empower employees to take responsibility for their actions and contribute to the organization’s sustainability goals can have a positive effect on their self-esteem which could enhance EGBs. Li et al. (2020) state that GTLs recognize employees’ unique qualities and requirements and provide individualized support and direction. This can help employees feel valued and respected, boosting their self-esteem and encouraging them to engage in environmentally conscious behaviors. Öğretmenoğlu et al. (2022) studied the impact of GTL on green creativity and the mediating effects of green organizational citizenship behaviors in a hospitality context. Their results indicated that GTL positively influenced employees’ green creativity and organizational citizenship behaviors. The underlying self-esteem potentially supports value congruence and green identity, which might be attributed to employees’ green behaviors, although not explicitly measured. Peng et al. (2019) elaborated upon self-esteem that it can indeed act as a mediator wherein it inspires and motivates followers to achieve their fullest potential and exceed their own expectations. Concomitantly, we hypothesize.

*H3*: FSE significantly and positively mediates the association between GTL and EGB during crises.

#### 2.3.4 The moderating role of EWE in the GTL-FSE link

GTLs inspire and motivate their employees by establishing a clear vision for a sustainable future, emphasizing the significance of environmental stewardship, and fostering innovation and green practices. Decuypere and Schaufeli (2020) have highlighted the role of work engagement in enabling leadership to produce desirable outcomes. According to Begum et al. (2022), GTLs demonstrate a strong commitment to sustainability and cultivate a sense of shared responsibility among team members by leading by example with a direct effect on employee engagement because it fosters a sense of purpose and meaning in the workplace. While elaborating upon the role of person-organization interaction in inducing EGBs, Mi et al. (2020) emphasized a need for person-organization fit (a key facet of EWE) in electrifying EGBs. Tao et al. (2022) discussed that employee engagement expands to employees’ emotional and intellectual commitment to the organization and its objectives’ wherein engaged employees are more likely to be innovative, productive, and loyal to their companies. Isserow (2023) emphasizes that it is crucial to determine how employees view themselves and their contributions to the organization as it leads the employees to recognize their abilities.

Busari et al. (2020) stated that employees may feel overwhelmed, anxious, or uncertain about their employment security and future prospects during a crisis, putting their self-esteem at risk. By fostering a positive and supportive work environment, recognizing individual accomplishments, and providing constructive feedback, GTLs can help boost employee self-esteem. According to Suliman et al. (2023), EWE could profoundly amplify the instrumentality of GTL by fostering an environment where employees feel valued, supported, and motivated to contribute to the organization’s sustainability initiatives. This sense of purpose and belonging can increase job satisfaction, performance, and commitment to the company’s mission and values (Suliman et al., 2023). Yücel (2021) elaborates on how SMEs’ employee engagement becomes even more crucial during times of crisis. Crises frequently bring about uncertainty, stress, and dread, which can have a negative effect on employee morale and output. GTLs can mitigate these effects by maintaining open communication, providing support and resources, and empowering employees to assume responsibility for their roles in responding to the crisis. Du and Yan (2022) added that by cultivating a sense of unity and shared responsibility, GTLs can assist employees in navigating difficult times and sustaining their engagement levels. GTL can possibly impact employee self-esteem by recognizing and rewarding their efforts, providing opportunities for growth and development, and fostering a collaborative and innovative work environment. Consonantly, we hypothesize.

*H4(a)*: EWE moderates the relationship between GTL and FSE.

#### 2.3.5 The moderating role of EWE in the FSE- EGB link

According to Costantini et al. (2019), self-esteem enhances a person’s overall perception of self-worth and personal worth; a high sense of self-worth enhances employee confidence, abilities, challenge ability, and maintains a positive outlook even in trying circumstances whereas low self-esteem can result in feelings of inadequacy, self-doubt, and a lack of motivation, which can have a negative impact on an employee’s performance, EWE could emerge as a panacea in such circumstances. Kwon and Kim (2020) stated that EWE is an employee’s emotive commitment and involvement with the organization and its objectives’ wherein engaged employees are more likely to be productive, motivated, and committed to their work, resulting in improved overall performance and job satisfaction. Raza et al. (2021) stated that self-esteem, employee engagement, and green behavior might pose a possible interconnection, as employees who feel good about themselves and are engaged at work are more likely to engage in pro-environmental actions. Employees with high self-esteem are more likely to be engaged in their work because they feel valued, competent, and capable of making significant contributions to the organization, which become manifold under higher EWE. Ansari and Irfan (2023) and Ansari et al. (2021) also found positive contextual effects of EWE in fostering EGBs through enabling the mediating psychological mechanisms like FSE.

During a crisis, FSE and EWE take on an even greater significance. Kuknor and Bhattacharya (2021) explained that a crisis can generate uncertainty, tension, and anxiety, which can have negative effects on the mental health and well-being of employees. In such circumstances, employees with a healthy sense of self-worth are better equipped to overcome obstacles and maintain their commitment to the job, and EWE could nicely augment it. Lin and Chen (2021) stated that organizations must continue to promote green behavior among their employees, as it can contribute to their long-term resilience and success. Even during difficult times, employees with high self-esteem augmented with high work engagement are more likely to recognize the importance of environmental sustainability and implement green practices at work (Raza et al., 2021). Following these assertions, we hypothesize.

*H4(b)*: EWE moderates the association between FSE and EGBs.

## 3 Research methodology

### 3.1 The measurement scales

The scales used to operationalize the constructs of this study have been adapted from previous studies. GTL has been operationalized through six items adapted from Azim et al. (2019). SME employees were asked to assess their leaders on the various aspects of GTL. A sample item included: “Our leader stimulates the organization members to think about green ideas.” Six items from Seppälä et al. (2009) were adopted to measure EWE. Employees were asked to self-report their work engagement on three dimensions. A sample item contained: “At my work, I always persevere, even when things do not go well.” To measure FSE, the scale used by Rajlic et al. (2019) has been adapted. The scale comprised six items where employees were asked to report their perceived levels of self-esteem. A sample item included: “I believe that I make valuable contributions.” Finally, six items obtained from Mahmud et al. (2023) were adapted for measuring EGBs. Respondents have been inquired about their display of green behaviors during times of crisis. A sample item included: “I try to engage in behaviors and initiatives that reduce social, economic, and environmental footprints during a crisis.” The responses for all the measurements were recorded on a 5-point Likert scale format, ranging from 1 (strongly disagree) to 5 (strongly agree). The adaptations mostly took place in question phrasings. The scale items were translated into Arabic language. Afterward, two academicians who were experts in these domains assessed the translated texts. A pilot test with 30 participants was conducted to determine whether they were applicable and appropriate.

### 3.2 Sampling and data collection

The sampled population of this study comprised employees working in SMEs in Saudi Arabia. SMEs in Saudi Arabia are businesses with fewer than 249 employees and SAR 200 million in annual revenue. Approximately 37% of the workers at these SMEs, which employ roughly 85% of them, are sole entrepreneurs. 74% of these SMEs are employed in trade and construction (Alenazi and Alanazi, 2023). The determination of sample size hinges on various factors such as normality, missing patterns, and model complexity (Wolf et al., 2013). Besides, as Hair et al. (2018) recommended 15 observations per independent variable, the appropriate sample size would be 90 observations. Finally, Cohen (1992) suggested that the sample size should be greater than 150 participants to gain a (i.e., 1-β) which is power of 80%, which leads to restrict a tendency of a type 2 error of 20% (i.e., β), with an expected medium effect size of 15% at an α equal to 5%. The final sample size used in this study (n = 232) has been more than the suggested minimum as per all these criteria.

We employed convenience sampling, a nonprobability sampling technique widely employed in qualitative and quantitative research because of its many benefits, including simplicity of use and proven effectiveness (Etikan, 2016; AlShammre et al., 2023). The convenience sampling technique has been extensively employed in similar studies in leadership (Dhar, 2016). We coordinated with the director of the SME department at the Chamber of Commerce, who helped us distribute the questionnaires to the relevant informants in the SMEs. Before launching the full-scale survey, the quality of the measurement scales was ascertained through a pilot survey (Rowley, 2014), and the results were quite supportive (See Table 1). The Cronbach’s alpha for the FSE was very high, which may indicate redundancy. A correlation test was performed for each FSE item, and all correlations were less than 0.5, indicating no redundancy issue.

The questionnaire has been distributed via email in coordination with the director of the SMEs department at the Chamber of Commerce to business owners or representatives in the SMEs to collect data from their employees. Out of the contacted, 250 informants participated in the survey. After removing responses with inadequate data (Newman, 2014; Hair et al., 2018), 232 valid responses were retained for the subsequent analysis.

**Table 1:** The pilot study results (*n* = 30).

Construct	Items	Cronbach alpha	Items total correlation
Green transformational leadership	GTL1	0.90	0.94^**^
	GTL2		0.98^**^
	GTL3		0.84^**^
	GTL4		0.80^*^
	GTL5		0.92^**^
	GTL6		0.89^**^
Employee engagement	EWE1	0.78	0.86^**^
	EWE2		0.92^**^
	EWE3		0.94^**^
	EWE4		0.94^**^
	EWE5		0.92^**^
	EWE6		0.94
Self esteem	FSE1	0.97	0.86^**^
	FSE2		0.92^**^
	FSE3		0.95^**^
	FSE4		0.94^**^
	FSE5		0.92^**^
	FSE6		0.94^**^
Green employee behavior	EGB1	0.93	0.91^**^
	EGB2		0.85^**^
	EGB3		0.90^**^
	EGB4		0.85^**^
	EGB5		0.84^**^

**A *p*-value of less than 0.01 is used.

### 3.3 Data analysis

Following Anderson and Gerbing’s (1988) and Ali et al.’s (2023), the analyses were performed in three steps. Performing descriptive statistics was the first step, then computing the measurement model to adjudge validity and reliability, and finally, testing the structural model to see if it could be considered appropriate to test the hypotheses. The analyses to ascertain the quality of the measurement scales and perform hierarchical regression in order to appraise the hypothesized associations have been performed using SPSS 23, macro V4 model 58, and Amos 24. The relevant results stemming from these analyses are presented in the next section.

## 4 Results

### 4.1 Sample profile

Table 2 shows the profile of the participants. The proportions of men and women were almost equivalent. Most participants, 54.3%, were aged between 37 and 45 years. 47.8% held bachelor’s degrees, and 54.3% held 6 to 10 years’ experience.

**Table 2:** Participants’ profile.

DV	*F* (*n* = 250)	(%)
*Gender*		
Men	118	50.9
Women	114	49.1
*Age*		
25 to 36 years	41	17.7
37 to 45 years	126	54.3
46 and above	65	28.0
*Educational level*		
Degree of Bachelor	111	47.8
Master	70	30.2
Doctoral	51	22.0
*Experience*		
3–5 years	22	9.5
6–10	126	54.3
10 years and more	84	36.2

DV; demographic variables, F; frequency; %; percent.

### 4.2 Assessment of the measurement model

#### 4.2.1 Assessment of reliability and multicollinearity of the items

Recommendations from Hair et al. (2018) were followed to validate the model’s measurements by assessing factor loadings (for indicator reliability), composite reliability and Cronbach Alpha (for constructs level reliability), AVE (for convergence validity), and HTMT values (for discriminating validity). Only the statistically significant items with factor loadings above 0.5 were retained. Multicollinearity was checked through the VIF values. Since the VIF values of all significant items were less than 5, therefore multicollinearity has not been deemed a relevant issue (Table 3).

**Table 3:** Factor loading and VIF values for the items.

Construct	Items	F.L.	VIF
Employee green behavior during crises (EGBs)	EGB1	0.50	1.15
	EGB2	0.86	2.92
	EGB3	0.75	2.01
	EGB4	0.77	2.10
	EGB5	0.84	2.80
Followers’ self-esteem (FSE)	FSE1	0.79	2.11
	FSE2	0.62	1.74
	FSE3	0.78	1.76
	FSE5	0.83	3.03
	FSE6	0.82	2.35
Employee work engagement (EWE)	EWE1	0.77	2.01
	EWE2	0.83	2.97
	EWE3	0.88	3.83
	EWE4	0.89	3.36
	EWE5	0.78	3.42
	EWE6	0.83	3.65
Green transformational leadership (GTL)	GTL1	0.56	1.72
	GTL2	0.81	2.33
	GTL3	0.83	2.57
	GTL4	0.82	2.10
	GTL5	0.92	3.78
	GTL6	0.82	3.03

#### 4.2.2 Reliability and validity at the construct levels

The internal consistency reliability at the construct level has been assessed through Cronbach Alpha and Composite Reliability measures using a 0.7 threshold suggested by Hair et al. (2022). All four constructs were found to be reliable. The average variance extracted (AVE) value was used to assess the convergent validity of the constructs, using the 0.5 benchmarks suggested by Hair et al. (2022). Sufficient convergent validity has been observed in all four constructs. The discriminatory validity of the measures was assessed using the Fornell and Larcker (1981) criteria. The square root of AVE for each construct has been found to be its correlation with all other constructs employed in the model, thereby indicating sufficient discriminant validity. Relevant statistics are contained in Table 4.

**Table 4:** Means, standard deviations, correlations, reliability and validity estimates (*n* = 232).

Constructs	Mean	S.D.	α	C.R.	A.V.E.	1	2	3	4
1. EGB	3.37	0.84	0.86	0.88	0.65	***0.81***			
2. GTL	3.91	0.87	0.91	0.92	0.75	0.69	***0.86***		
3. FSE	3.86	0.83	0.88	0.89	0.68	0.71	0.72	***0.83***	
4. EWE	3.57	0.84	0.88	0.88	0.62	0.52	0.46	0.55	***0.79***

GTL, green transformational leadership; green behavior during crises, SE, follower’s self-esteem; EWE, employee work engagement; α, alpha chronbach; C.R., composite reliability; AVE, average variance.

### 4.3 Assessment of the structural model

#### 4.3.1 Model’s goodness of fit

**Figure 2:**
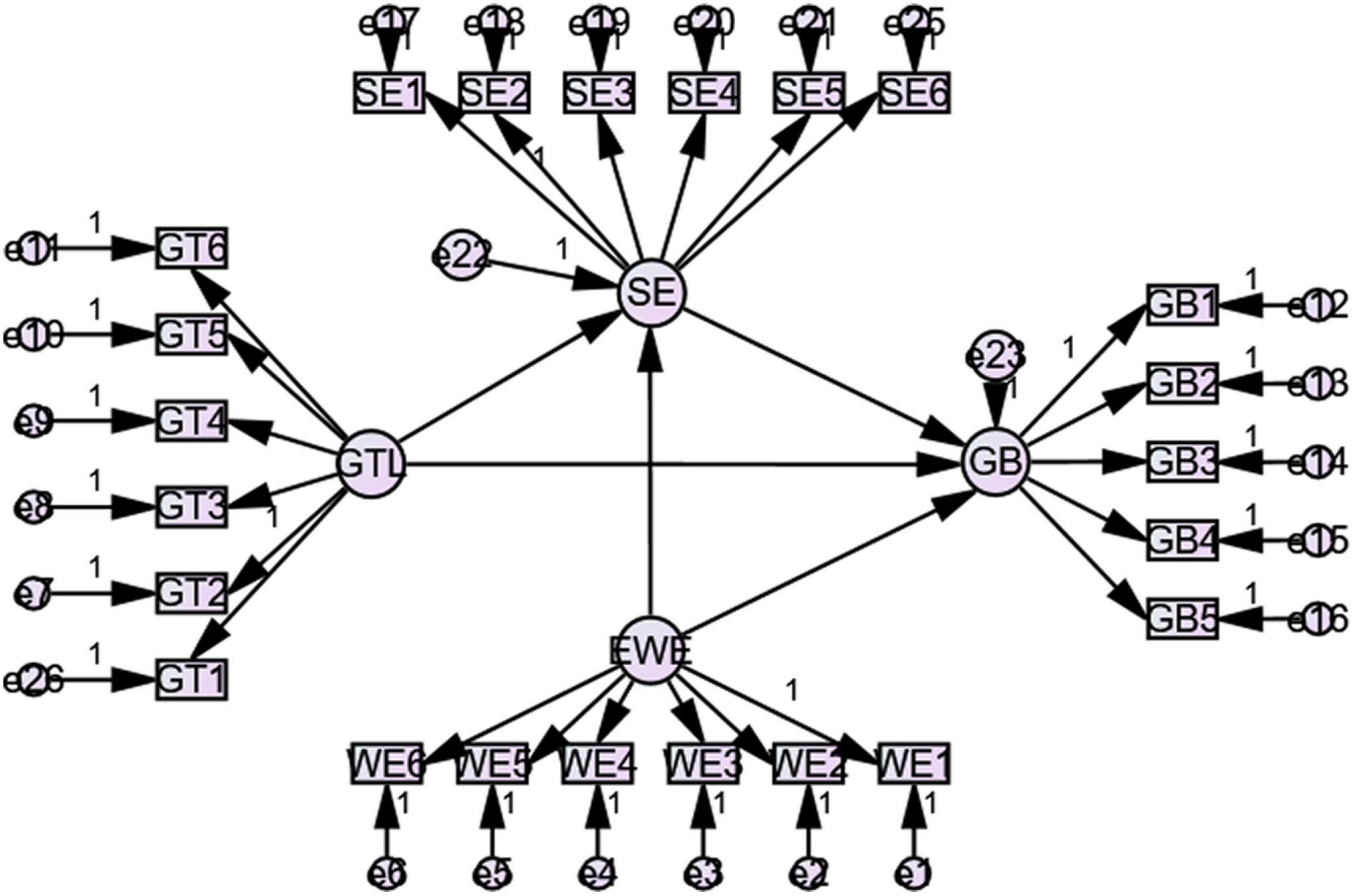
The structural model.

Following Roussel and Wacheux (2005), absolute, incremental, and parsimony indices were calculated to assess the model’s goodness of fit. For this purpose. These indices are presently thought to be the most stable, least susceptible to model complexity and sample size, and statistically most robust (Hu and Bentler, 1999; Barrett, 2007; Kline, 2011). AMOS 24 has been used to calculate these indices. As shown in Table 5, all of these indices showed a sufficient fit except GFI, which remained slightly below par. However, according to Wang et al. (1996), GFI mainly uses the information of the covariance matrix, which is not steady in small samples and gradually develops stable with the growth of sample size. However, our primary focus has been the SRMR, as Henseler et al. (2015) suggested, which showed an acceptable value of less than 0.08 (Hair et al., 2018; Figure 2).

**Table 5:** Model fit indices.

Fit indices	Estimates	Acceptable level
Chi-square	423.55	
Degree of freedom (d.f)	186	
*p*	0.00	>0.05
Normed Chi-square (CMIN/DF)	2.28	<3.00
Goodness-of-Fit Index (GFI)	0.86	≥0.90
Comparative Fit Index (CFI)	0.95	≥0.95
Root Mean Square Error Approximate (RMSEA)	0.07	<0.08
Standardized Root Mean Square Residual (SRMR)	0.04	<0.08

#### 4.3.2 Hierarchical regression analysis

Initially, the independent variables exhibited a multicollinearity issue (the independent variables being correlated). In order to deal with this problem, the independent variables have been centered on reducing structural multicollinearity. Table 6 contains the relevant statistics. After treatment, tolerance values are significantly more than 0.2, and their VIF scores are significantly less than 5 (Bowerman and O’Connell, 1990).

**Table 6:** Tolerance and VIF before and after centering the independent variables.

	Before centering		After centering	
Constructs	Tolerance	VIF	Tolerance	VIF
2 GTL	0.117	8.520	0.954	1.048
3. FSE	0.068	14.615	0.953	1.049
4. EWE	0.089	11.224	0.958	1.044

Relevant statistics of the alternative models calibrated through hierarchical regression are reported in Table 7. The second model (M2) captures the direct impact of GTL on FSE. A strong positive link between GTL and FSE could be witnessed here (β = 0.93, *p* < 0.001). Model 3 adds EWE as the moderator which results in a drop in the coefficient of GTL from (β =0.93, *p* < 0.001) in M2 to (β =0.40, *p* < 0.001) in M3, with the moderating role of EWE also being found significant to (β =0.60, *p* < 0.01). Model 5 (M5) evaluated the direct link between GTL and EGB during crisis, a positive association has been corroborated here (β = 0.95, *p* < 0.001). Model 6 adds FSE to the system, and it can be seen that the GTL’s direct impact is reduced (β =0.21, *p* < 0.001) whereas the FSE coefficient is increased to (β =0.79, *p* < 0.001), reflecting a positive mediation effect. Model 7 (M7) adds the moderating influence of EWE to the system. The results reflect a significant positive direct association between GTL and EGB (β =0.13, *p* < 0.05), in addition to significant roles played by FSE (β =0.51, p < 0.001) and EWE (β =0.37, *p* < 0.01).

**Table 7:** Hierarchical regression results.

Variables	Follower’s self-esteem (FSE)			Employee green behavior during crisis (EGB)			
Model	M1	M2	M3	M4	M5	M6	M7
Intercept	4.07^***^	0.48	−0.07	4.51^***^	0.87^***^	0.49^***^	0.28^*^
Gender	−0.09	−0.05	−0.03	−0.08	−0.05	−0.01	−0.01
Age	0.18^*^	0.03	0.03	0.14	−0.01	−0.03	−0.02
Education	−0.11	0.02	0.03	−0.14	−0.01	−0.02	−0.01
Experience	−0.24^*^	−0.03	−0.03	−0.23^**^	−0.02	0.00	−0.01
GTL		0.93^***^	0.40^***^		0.95^***^	0.21^***^	0.13^*^
FSE (Mediator)						0.79^***^	0.51^***^
EWE(Moderator)			0.60***				0.37^***^
*R* ^2^	0.05	0.88	0.95	0.04	0.84	0.91	0.93
Δ*R*^2^	0.05	0.83	0.06	0.04	0.80	0.07	0.01
F	2.79	319.66^***^	510.55^***^	2.64^*^	24011^***^	399.5^***^	396.7^***^
Df	227	226	225	227	226	225	224

M, Model. ^***^p < 0.001, ^**^p < 0.01, ^*^p < 0.05.

#### 4.3.3 Hypothesis testing

The approach proposed by Preacher et al. (2007) was utilized to test the hypothesized model using PROCESS Macro V4. First, we looked at the direct and moderated associations envisaged by Hypotheses 1, 2, 3(a&b), and 4, and then we assessed the mediation effects and the wholesome moderated mediation model specified by Hypothesis 3 using PROCESS macro-Model 58. The data were mean centered before testing the model, as Aiken and West (1991) advised.

Table 8 displays hypothesis testing results of H1 and H4(a). A significant association between GTL and FSE (β =0.22, *t* = 2.40, *p* < 0.05), moderated through EWE has been empirically substantiated (β =0.04, *t* = 2.15, *p* < 0.01).

**Table 8:** Regression results for the direct effects – indirect effect of mediation and moderation on follower’s self-esteem and green behavior during crisis.

Direct relationships	β	SE	Lower level CI	Upper level CI	*T*-value	Direct relationships	β	SE	Lower Level CI	Upper level CI	*T*-value
Gender	−0.03	0.03	−0.08	0.03	−0.85	Gender	0.00	0.03	−0.06	0.06	−0.02
Age	0.03	0.02	−0.02	0.07	1.22	Age	−0.02	0.02	−0.07	0.03	−0.82
Education	0.03	0.02	−0.01	0.06	1.33	Education	−0.02	0.02	−0.06	0.02	−0.77
Experience	−0.03	0.03	−0.08	0.02	−1.16	Experience	−0.01	0.03	−0.06	0.05	0.30
GTL → FSE	0.22	0.09	0.04	0.40	2.4*	FSE → EGB	0.28	0.12	0.04	0.51	2.33*
GTL*EWE → FSE	0.04	0.02	0.01	0.08	2.15*	FSE*EWE → EGB	0.06	0.02	0.01	0.05	2.49*

Indirect relationship	Effect	Boot SE	Lower-Level CI	Upper-Level CI	Indirect relationship	
GTL → SE → EGB	0.74	0.08	0.58	0.88	GTL → FSE → EGB	
*F*	445.28				*F*	355.99
*R* ^2^	0.93				*R* ^2^	0.93

*n* = 232, ^**^p < 0.01; ^*^p < 0.05.

**Figure 3:**
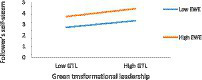
The impact of GTL on FSE at high and low levels of EWE.

To reflect the moderation effects, the association between GTL on FSE at high level and low level of EWE has been drawn, at high level (one standard deviation above the mean) and low level (one standard deviation below the mean) in accordance with prior ideas (Aiken and West, 1991). As demonstrated in Table 9 and Figure ***3***, the relationship between GTL and FSE is higher with a higher EWE (*β* = 0.43, *t* = 9.40, *p* < 0.001) as compared to a low EWE (β = 0.35, *t* = 7.46, *p* < 0.01).

**Table 9:** The impact of green transformational leadership on followers’ self-esteem at high and low levels of work engagement.

EWE	Effect	SE	*t*-values	Lower level CI	Upper level CI
-SD	0.35	0.05	7.46***	0.26	0.45
Mean	0.39	0.04	9.02***	0.31	0.48
+SD	0.43	0.05	9.40***	0.34	0.52

**Figure 4:**
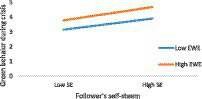
The effect of FSE on EGB at high and low levels of EWE.

Table 8 also includes the findings about Hypotheses H2 and H4(b). A significant positive link between FSE and EGBs has been corroborated (β =0.28, *t* = 2.33, *p* < 0.05), as well as the moderation of EWE in this association (β = 0.06, *t* = 2.49, *p* < 0.05). As can be witnessed in Table 10 and Figure 4, the relationship between FSE and EGB is lower at low levels of EWE (β =0.44, *t* = 5.52, *p* < 0.01) and becomes stronger at higher levels of EWE (β =0.54, *t* = 7.44, *p* < 0.01).

**Table 10:** The effect of FSE on EGB at high and low levels of EWE.

EWE	Effect	FSE	*T*-value	Lower level CI	Upper level CI
−SD	0.44	0.08	5.82^***^	0.29	0.59
Mean	0.49	0.07	6.82^***^	0.35	0.63
+SD	0.54	0.07	7.44^***^	0.39	0.68

The results also confirm the indirect association between GTL and GB through FSE (β =0.74, 95% CI [0.58, 0.88]), corroborating H3. We can conclude that SE partially mediates the relationship between GTL and GB. Additionally, the moderated mediation effect was also assessed. The indirect effect between GTL and EGB through FSE at the mean of EWE and one standard deviation above and below are displayed in Table 11. As expected, the indirect link is lower when the EWE is lower (β =0.16, 95% CI [0.07, 0.28]), and it is increased to (β =0.23, 95% CI [0.13, 0.37]) at the high level of EWE.

**Table 11:** Conditional indirect effect of GTL on EGB via FSE at low and high valued of EWE.

		Test for the conditional indirect effect with moderation		
EWE	Indirect effect	Boot FSE	95% CI lower bound	95% CI upper bound
−SD	0.16	0.05	0.07	0.28
Mean	0.19	0.06	0.10	0.72
+SD	0.23	0.06	0.13	0.37

## 5 Discussion

Hypothesis one, which postulated a direct association between GTL and FSE, has been empirically substantiated by the results of this study. It is in line with some of the previous studies. For example, Chen et al. (2022) also found that there was a substantial connection between transformational leadership and self-esteem. They concluded that FSE could be improved through transformational leadership, particularly when it is centered on green, responsible behaviors. Additionally, Hameed et al. (2021) studied the link between GTL and FSE and found a favorable association between GTL and enhancement in employees’ sense of value and self-esteem, consequently affecting the display of green behaviors. Begum et al. (2022), Khan (2023), and Srour et al. (2020) also found a positive association between transformational leadership and desirable employee behaviors in different geographical contexts. Gupta and Singh (2020) posited that transformational leaders can provide the necessary support and guidance during a crisis to help employees navigate challenging situations, ultimately boosting their self-esteem. Edmondson (2019) stated that transformational leadership affects self-esteem during a crisis by fostering a sense of psychological safety among employees (Edmondson, 2019). This is achieved through open communication, empathy, and trust, which allows team members to express their concerns and ideas without fear of negative consequences. By creating a psychologically safe environment, transformational leaders can help employees maintain their self-esteem during difficult times (Gupta and Singh, 2020). According to Chen et al. (2022), GTL can impact self-esteem during a crisis by promoting resilience among employees.

Hypothesis two, which postulated a direct positive association between FSE and EGBs, has also been empirically supported, aligning with some previous studies. During crises like the COVID-19 epidemic, employment uncertainty, financial hardship, and work routine changes can lower employees’ self-esteem. Srivastava and Gupta (2022) observed that employees with higher self-esteem were more adaptable to crisis-related stressors and remained committed to green behaviors. High self-esteem gives people more confidence to handle challenges and adapt to new conditions. Crisis-related pressures may truncate green behavior in employees by lowering their low self-esteem. According to Gkargkavouzi et al. (2019), people with low self-esteem are more likely to struggle in the face of environmental hazards, which can reduce their sense of personal responsibility and their likelihood of engaging in green behaviors. During times of crisis, people with low self-esteem may prioritize their immediate wants and worries over long-term environmental goals. Balaji et al. (2019) assert that SMEs confront financial restrictions, supply chain interruptions, and changing market needs during crises. In such cases, business owners’ and employees’ self-esteem can affect their capacity to adopt and maintain green practices. Low-esteem individuals may feel overwhelmed by their circumstances and choose short-term survival over long-term sustainability. Fachrunnisa (2022) concluded that such an approach could lead to the abandoning of green projects in favor of more urgent concerns, worsening environmental challenges, and threatening the business’s future. Low self-esteem can cause short-term thinking and decision-making.

Hypothesis 3, which put forth a positive mediation effect of FSE in the GTL-EGB link, also got sufficient empirical support from the findings of this study, which is in consonance with previous literature. Ahmad et al. (2021) demonstrated that FSE as a mediator for GTL’s influence on EGB signifies the possibility for GTL to augment FSE, which may trigger EGBs. Öğretmenoğlu et al. (2022) also revealed that employees with a high sense of self-worth are more likely to have confidence in their talents, take on demanding jobs, and persevere in the face of adversity. They are also more likely to be proactive, looking for possibilities for development and progress and accepting responsibility for the acts they do. Because of this, there is the potential for enhanced productivity as well as improved innovation and sustainability performance. Peng et al. (2019) elucidate that workers with healthy self-esteem are actively involved in their jobs and devoted to the sustainability gains of the organization. They are more likely to have a feeling of ownership and responsibility for their jobs, which ultimately leads to a better sense of accountability and the desire to contribute to the success of the organization, all of which transcends to the shoulders of a GTL.

The findings of this study also yielded strong empirical support for Hypothesis 4 (a), which stated a positive moderation effect of EWE in the GTL-FSE linkage, which means that when EWE is high, the impact of GTL on FSE is likely to be greater. As they have a strong connection to the organization and its mission, engaged employees are more likely to be receptive to the values and objectives promoted by green transformational leaders. This, in turn, could catalyze employees’ perceptions of the manfulness of their work and the ensuing contributions to the greater good. This finding is consistent with previous literature. Tao et al. (2022) explained that when employee engagement is minimal, the impact of GTL on FSE may be diminished. Employees who are disengaged may not completely embrace the eco-friendly initiatives and values promoted by their leaders, resulting in a weakened relationship between leadership and self-esteem. According to Begum et al. (2022), to maximize the positive impact of GTL on FSE in such circumstances, it is essential for organizations to improve EWE. Additionally, Du and Yan (2022) found that engaged employees are more likely to participate actively in sustainability initiatives, exchange ideas, and collaborate with co-workers to achieve the organization’s environmental objectives. This collaborative endeavor can contribute to the overall success of GTLs, resulting in a greater impact on FSE.

Finally, hypothesis 4(b), which assumed a positive moderation effect of EWE on the FSE-EGB linkage, also found adequate empirical support, which is in line with some of the previous studies. According to Ansari and Irfan (2023), the relationship between FSE and their behavior in times of crisis is significantly influenced by employee engagement. In times of crisis, employees are required to demonstrate adaptability, resilience, and sound decision-making in the face of unprecedented obstacles. Employees who are emotionally invested in their work and are committed to the organization’s success demonstrate positive behaviors and contribute to overcoming the crisis. Raza et al. (2021) elaborated that when employees feel valued, supported, and connected to the organization’s goals, their self-esteem tends to increase. Their sense of self-worth can positively affect their (green) behavior during a crisis, as they are more likely to remain composed, solution-oriented, and focused. Kuknor and Bhattacharya (2021) found that engaged employees with a high sense of self-worth are likelier to take initiative, collaborate effectively with co-workers, and demonstrate resilience in the face of adversity. In addition, the positive relationship between EWE and green behavior during a crisis has been delineated by their study.

## 6 Conclusion

### 6.1 Theoretical contributions

The findings of this study contribute to the advancement of scholarly discourse on EGBs in many ways. EGB during crises is in its infancy, and scant studies so far have examined GTL as a key antecedent of EGBs during crises. By empirically substantiating that GTL directly as well as indirectly enhances EGBs during crises, this research makes an important contribution to initiating and/or nurturing discussion and debate in this domain. It adds strength to the efficacy of transformational leadership theory, social exchange theory, and social cognition theory in explaining the dynamics of EGBs through the lens of leadership-follower relationships. The empirical substantiation of the impact of GTL on EGBs through the mediation of FSE and moderation of EWE reveals an important nexus that has not been discussed holistically in the previous literature. Additionally, the new-fangled model sets an agenda for future research to enrich the rigor of this baseline model by integrating diverse mediating and contextual conditions. Furthermore, findings girdle the conditions under which the GTL could produce better results in maturing EGBs during crises. Thus, the study provides a deeper understanding of the boundary conditions of GTL affecting EGBs. Another unique contribution of this study emanates from its usage of Middle Eastern data that not only provides novel insights into a unique socio-cultural milieu but could also swell the generalizability of the etic theories developed in the West to other cross-cultural contexts.

### 6.2 Practical implications

Organizations, especially SMEs must appreciate the efficacy of GTL, FSE, and EWE in producing desirable (green) behaviors, particularly during times of crisis, and should take concrete measures to enhance them to boost EGBs. Adequate nurturing and integration of these essential conditions may have a substantial effect on an organization’s capacity to overcome obstacles, maintain productivity, and ensure long-term sustainability. GTL is essential for navigating organizations through uncertainty and change during times of crisis. Besides inspiring and motivating their followers, GTLs must strongly integrate green orientation in their decision-making. By doing so, they can assist organizations in adapting to new market conditions, adhering to green regulations, and maintaining a positive reputation among stakeholders. Encouraging EGBs can aid organizations in reducing their carbon footprint, lower operational expenses, and enhance overall productivity. Fostering enabling mechanisms like FSE and EWE could help GTLs enhance their followers’ readiness and efficacy for such contributions.

The post-pandemic era witnessed a massive change in organizations and leadership. GTL promotes green practices within an organization which entails inspiring and motivating employees to embrace green behaviors, participate in green initiatives, and contribute to the organization’s overall sustainability objectives (Yaqub et al., 2023). Organizations should, therefore, invest in nurturing and enabling GTL competencies (Ali et al., 2023). This could be accomplished through efficacious training and development programs instituted at all levels (top leadership, managers, employees). Besides, leaders should inspire and motivate their employees to adopt and implement green behaviors through mentoring and orchestrating strong social discourse. In addition, GTLs should involve their employees in the decision-making processes pertaining to corporate green initiatives to cement their sense of ownership for such initiatives (Yaqub et al., 2023). Organizations may also establish green teams or committees to encourage employee participation in sustainability initiatives. In addition, organizations should recognize and reward employees who exhibit green consciousness, behaviors, and advocations to contribute to the organization’s sustainability objectives. This can be accomplished through a variety of methods, including public recognition, monetary incentives, and additional benefits. By recognizing and rewarding green employee actions, managers can reinforce the significance of green behavioral practices and encourage others to do the same. Finally, managers should set an example by displaying a strong personal commitment to green-conscious practices. This may involve reducing energy consumption, recycling, and utilizing favorable green products and other corporate citizenship behaviors. By demonstrating their personal commitment to green practices, leader can motivate their employees to do the same and cultivate and/or strengthen a green culture which proves to be quite handy during exigent times.

### 6.3 Limitations and future research suggestions

A study’s limitations (or constraints) underscore the domain(s) in which future investigations can augment our comprehension of the subject matter. Although this study offers some cardinal contributions to the understanding of the dynamics of the maturing of EGBs during crises, it is important to acknowledge certain limitations. First, the generalizability of the results may be constrained by the specific sample and context in which it is based. The sample for this study comprised exclusively SMEs operating in Saudi Arabia, limiting the generalizability of its results. Subsequent investigations should replicate this study by employing heterogeneous samples from other demographic, temporal, geographical, and cultural contexts to augment the generalizability of the results. Second, this study employed a cross-sectional design, a method that captures a momentary representation of the associations among constructs at a particular instance. Longitudinal or experimental methodologies may offer a more comprehensive understanding of the causal associations over time. To enhance the quality of the findings in the future, this study recommends multi-method quantitative research with a longitudinal time horizon. Third, the research has depended on self-reporting measures, which are susceptible to common methods and potential response biases. Subsequent investigations should consider the inclusion of objective metrics or diverse data sources to augment the veracity of outcomes. Fourth, data used in this investigation were obtained from a single source, with a predominant reliance on the subjective evaluations and self-appraisals of GTL, FSE, ESE, and EGB. The integration of multiple data sources can yield a more comprehensive understanding of the relationships being examined.

The study demonstrates that EGBs during crises improve with the help of GTL, FSE and EWE. Future researchers could employ other mediators and/or mechanisms between GTL and EGBs. Additionally, exploring the role of moderating variables such as organizational culture, knowledge sharing, and perceived organizational support could also be possible. In addition, a multi-level perspective (e.g., macro, team, etc.) should be incorporated into future research to capture a more holistic view. Finally, exploring possible discrepancies in the associations between these constructs within disparate sociocultural settings could provide useful insights into the limitations and situational elements that influence these associations.

### 6.4 Conclusion

This study investigates the efficacy of GTL as a critical antecedent that could encourage, support, and enable employees to exhibit green behaviors during times of crisis. Specifically, the study examines two important mediating and moderating conditions that enable GTL to produce the desired effect on EGBs. It demonstrates that FSE as a psychological mechanism significantly mediates the GTL-EGB linkage. In addition, it elucidates that an enhancement in the EWE complements the efficacy of GTL in enhancing FSE, as well as FSE’s instrumentality in maturing EGBs during tempestuous times.

GTL signifies an unraveled commitment to ethics, sustainability, and social responsibility during exigent conditions. GTLs, while believing in the strong participation of their followers in the collective efforts to uphold green behaviors, need to use their idealized influence and inspiration motivation to enhance FSE and EWE, which may contrive just the right enabling conditions under which the efforts of GTLs to nurture EGBs during crises could produce desired results. The study empirically substantiates the assertion that employees with high self-esteem and superior work engagement are more receptive and responsive to their GTLs’ efforts to uphold green behaviors and practices during hard times. Therefore, SMEs, including their managers, leaders, and/or entrepreneurs, need to create enabling conditions where employees feel esteemed and highly engaged and invest in nurturing and/or enabling the GTL competencies.

## Data availability statement

The raw data supporting the conclusions of this article will be made available by the authors, without undue reservation.

## Ethics statement

Ethical review and approval was not required for the study on human participants in accordance with the local legislation and institutional requirements. Written informed consent from the participants was not required to participate in this study in accordance with the national legislation and the institutional requirements.

## Author contributions

WA: Conceptualization, Formal analysis, Investigation, Methodology, Software, Validation, Writing – original draft. MY: Conceptualization, Investigation, Methodology, Project administration, Resources, Supervision, Writing – review & editing.
